# Still to ARRIVE at adequate reporting of orthodontic studies involving animal models

**DOI:** 10.1093/ejo/cjae032

**Published:** 2024-07-15

**Authors:** Dihya Flitti, Nikolaos Pandis, Jadbinder Seehra

**Affiliations:** Department of Orthodontics, Faculty of Dentistry, Oral & Craniofacial Sciences, King’s College London, Floor 21, Guy’s Hospital, Guy’s and St Thomas NHS Foundation Trust, London, SE1 9RT, United Kingdom; Department of Orthodontics and Dentofacial Orthopedics, Dental School/Medical Faculty, University of Bern, Bern, Switzerland; Department of Orthodontics, Faculty of Dentistry, Oral & Craniofacial Sciences, King’s College London, Floor 21, Guy’s Hospital, Guy’s and St Thomas NHS Foundation Trust, London, SE1 9RT, United Kingdom; Centre for Craniofacial Development & Regeneration, Faculty of Dentistry, Oral & Craniofacial Sciences, King’s College London, Floor 27, Guy’s Hospital, Guy’s and St Thomas NHS Foundation Trust, London, SE1 9RT, United Kingdom

**Keywords:** ARRIVE guidelines, orthodontics, animal model

## Abstract

**Background:**

The ARRIVE 2.0 guidelines were introduced to improve the reporting of animal studies. The aim of this study was to assess the reporting adherence of orthodontic speciality animal studies in relation to ARRIVE 2.0 guidelines. Associations between the reporting and study characteristics were explored.

**Materials and method:**

An electronic database search was undertaken using Medline via PubMed (www.pubmed.ncbi.nlm.nih.gov) to identify studies meeting the eligibility criteria published between 1 January 2018 and 31 December 2023. Data extraction was performed in duplicate and independently. Descriptive statistics and frequency distributions for the responses to each checklist item were calculated. Mean values for adequate reporting per ARRIVE item were calculated. A sum score was calculated by adding the responses (0 = not reported, 1 = inadequate reporting, 2 = adequate reporting) per item and sub-questions. On an exploratory basis, univariable linear regression between summary score and study characteristics (year of publication, continent of authorship, type of centre, and number of authors) was performed.

**Results:**

Three hundred and eighty-four studies were analysed. Variability in the adequate reporting of the ARRIVE 2.0 guideline items was evident. In particular, in 32% of studies, there was a lack of reporting of the priori sample size calculation. Overall, the mean reporting score for the sample was 57.9 (SD 6.7 and range 34–74). There were no associations between score and study characteristics except for a weak association for year of publication with a small improvement over time (each additional year).

**Conclusions:**

The reporting of animal studies relevant to the speciality of orthodontics is sub-optimal in relation to the ARRIVE 2.0 guidelines. There was a tendency for the non-reporting of items pertaining to study sample size, eligibility, methods to reduce bias and interpretation/scientific implications. Greater awareness and reporting adherence to the ARRIVE 2.0 guidelines are required to reduce research waste involving animal models.

## Introduction

Despite the introduction of computer models and tissue and cell cultures within scientific research, studies involving animal models remain a viable and important research resource [1]. This is highlighted by the fact that in 2005 over twelve million animals were used for experiments in Europe [2]. Studies involving animal models are commonly utilized to assess the safety and efficacy of interventions, where the results are then extrapolated to understand the possible mechanism of action of the intervention in humans [3]. The validity of this approach has been questioned [4], as extrapolation of results on a 1:1 basis with human samples is hindered by variation in study design, bias, confounders, and innate differences in the underlying physiology between human and animal species. Furthermore, animal research is utilized when the results must be obtained by sacrifice of the animal and further histological investigation. The benefits of animal studies in orthodontics have been highlighted [5]. For instance, using the mice model, the use of local RANKL gene transfer has been reported to accelerate tooth movement and hence has the potential to reduce treatment duration [6].

It has been reported that animal studies suffer from poor reproducibility which can ultimately affect the validity and impact of the findings [7, 8]. Errors and incomplete reporting of key components of study methodology leading to heterogeneity between animal studies have been highlighted [9]. Importantly omissions in reporting hinder the translatability of animal research to humans [10, 11]. This is highlighted by the fact that the reported effects of an intervention in animal studies tend to be greater than those reported in human studies [12]. However, the lack of homogeneity in study design and data analysis precludes direct comparisons between both study types [12]. It is commonly accepted that Randomised Clinical Trials (RCTs) are considered the optimal study design to investigate clinical efficacy, effectiveness, and safety of interventions. Although the aims and objectives of animal studies may differ from those of randomized clinical trials, reporting of both study types should be subject to the same level of scrutiny and rigor [13].

To facilitate clear and transparent reporting of animal studies the ARRIVE guidelines were initially developed and introduced in 2010 [14] and subsequently updated and superseded by the ARRIVE 2.0 guidelines in 2020 [15]. The original guidelines consisted of 20 items whereas the updated version is split into ten essential and 11 recommended items. The authors of the updated guidelines state they ‘reordered items and split them into two sets based on their importance to assess the reliability of the study. There is no ranking within each set, items are ordered logically’ [15]. The updated ARRIVE 2.0 guidelines cover aspects such as study design, methodology, animal welfare, interpretation, and generalisability of results [15]. New items added comprise of stating the inclusion and exclusion criteria, protocol registration, analysis plan, and data access [15]. Previous studies assessing reporting adherence to the original ARRIVE guidelines have identified several areas of deficient reporting including items pertaining to a description of the housing and husbandry, information regarding the sample size calculation, and allocation of animals to experimental groups [16, 17]. The introduction of the new ARRIVE 2.0 guidelines has led to better reporting, but further improvement is still required [18].

As far as we are aware, an assessment of the reporting of animal studies published in the orthodontic literature has not been previously undertaken. The aim of this investigation was to assess the reporting adherence of animal studies relevant to the speciality of orthodontics in relation to ARRIVE 2.0 guidelines. Associations between the reporting of studies and study characteristics were explored.

## Materials and methods

### Eligibility criteria

Studies involving any type of animal model were eligible for inclusion in this assessment. Studies published in English were only sourced. Studies involving human participants, systematic reviews, randomized clinical trials, prospective studies, case reports, pilot studies, and protocols were excluded.

### Search for relevant articles

An electronic database search was undertaken using Medline via PubMed (www.pubmed.ncbi.nlm.nih.gov). One author (J.S.) performed a literature search of this database using medical subject headings and free-text terms related to ‘orthodontic’. The following search filters were applied in the search: articles published between 1 January 2018 and 31 December 2023, English language only and involving animal models. The title, abstract and methodology section of each study were screened for the reported use of animal models. The applicability of the study to the orthodontic speciality was agreed by discussion between two assessors (D.F. and J.S.).

### Selection and data extraction

The titles, abstracts, and full text of articles meeting the eligibility criteria were assessed independently by two assessors (D.F. and J.S.). Disagreements were discussed between both assessors (D.F. and J.S.) and resolved by a third assessor if required (N.P.). Data from each eligible animal study were extracted by both assessors (D.F. and J.S.) independently. Any disagreements were discussed between both assessors and resolved by a third assessor (N.P.). A standardized and pre-piloted data extraction form was used. Prior to data extraction, both assessors (D.F. and J.S.) individually undertook an initial pilot calibration. The results were discussed between both assessors (D.F. and J.S.). Any disagreements were resolved by discussion a third assessor (N.P.). 100% agreement was achieved.

At the study level, the following study characteristics were extracted: year of publication, journal title, continent of corresponding author (Europe, Americas and Asia, and other), number of authors, centre (single or multi), statistical significance of primary outcome (yes or no), and type of animal model. The reporting adherence of each animal study was assessed in relation to the ARRIVE 2.0 guidelines [15] (Supplementary Table I). This checklist consists of two parts: essential 10 and the recommended set (11 items), totalling 21 items. Further sub-type questions are required in 13/21 checklist items. The reporting of each item was assigned to one of three categories (0 = not reported, 1 = inadequate reporting and 2 = adequate reporting). To ensure consistency in the interpretation of each checklist item by the assessors, the pre-defined description of each item was directly referred to during data extraction. Furthermore, each item and their sub-type questions were scored based on applicability. Each article and supplementary data files were also screened to determine if the authors had explicitly stated if they had reported their study (yes or no) in relation to the ARRIVE guidelines.

### Statistical analysis

Descriptive statistics and frequency distributions for the responses to each checklist item were calculated. Mean values for adequate reporting per ARRIVE item were calculated and plotted using a radial plot. A sum score was calculated by adding the 0/1/2 per item response per question. On an exploratory basis, univariable linear regression between summary score and the following study characteristics was performed: year of publication, continent of authorship, type of centre, and number of authors. All analyses were conducted using the R programming language version 4.3.1 (Vienna, Austria).

## Results

**Figure 1.**
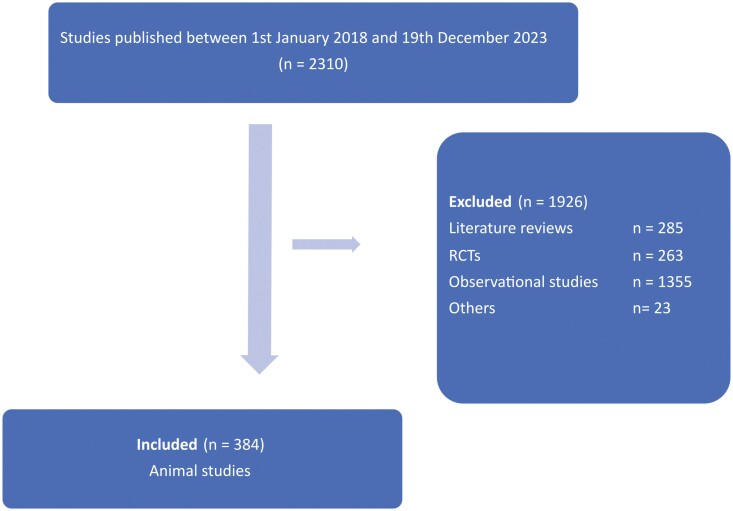
Flow diagram for study identification (N = 384). (*Others = could not retrieve full-text of article).

The initial search identified 2310 studies, of which 384 were deemed eligible for inclusion in this study (Fig. 1) (Supplementary Table II). The most frequent years of publication were 2020 (21.3%) and 2022 (21.1%). Corresponding authors of animal model studies were likely to be based in Asia and other countries (69.2%). Studies were typically multicentre (80.0%) and reporting a significant finding for the primary outcome. The median number of authors was 7 (IQR 4). The rat (61.9%) followed by mice (24.7%) were commonly used as the animal models (Table 1).

**Table 1.** Study characteristics (*N* = 384).

Variable	*N* (%)
**Year of publication**	
2018	71 (18.5)
2019	53 (13.8)
2020	82 (21.3)
2021	64 (16.7)
2022	81 (21.1)
2023	33 (8.6)
**Continent of corresponding author**	
Europe	44 (11.5)
Americas	74 (19.3)
Asia and other	266 (69.2)
**Number of authors**	
1	1 (0.3)
2	17 (4.4)
3	20 (5.2)
4	29 (7.5)
5	52 (13.5)
6	58 (15.1)
7	50 (13.0)
8	46 (11.9)
9	49 (12.6)
10	28 (7.3)
11	12 (3.1)
12	7 (1.8)
13	3 (0.8)
14	6 (1.6)
15	3 (0.8)
19	1 (0.3)
20	1 (0.3)
21	1 (0.3)
Median	7 IQR 4
**Centres**	
Single	76 (20.0)
Multi	308 (80.0)
**Statistical significance of primary outcome**	
No	13 (3.4)
Yes	371 (96.6)
**Type of animal model**	
Rat	238 (61.9)
Mice	95 (24.7)
Canine	19 (4.9)
Pig	7 (1.8)
Avarian	1 (0.3)
Bovine	5 (1.3)
Fish	1 (0.3)
Horse	1 (0.3)
Monkey	1 (0.3)
Guinea pig	1 (0.3)

**Figure 2.**
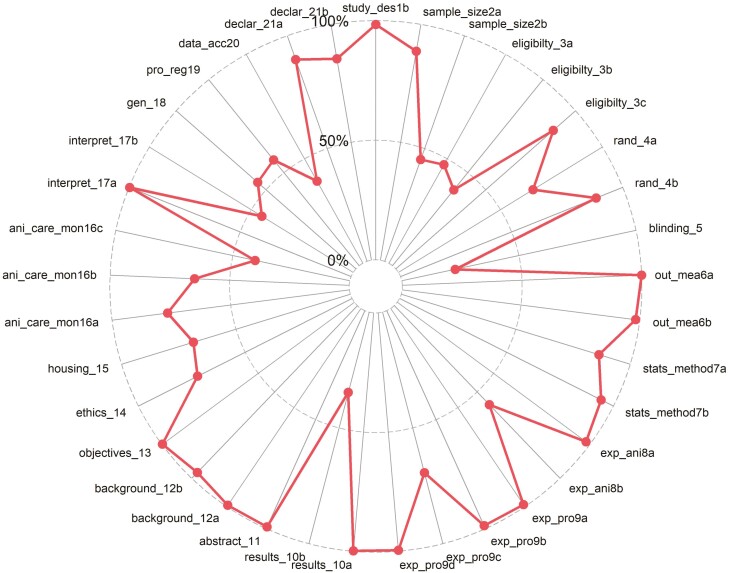
Radial plot displaying mean adequate responses per ARRIVE 2.0 checklist items.

The distribution of scores per category (0 = not reported, 1 = inadequate reporting, and 2 = adequate reporting) for the ARRIVE 2.0 items are presented in Table 2. Items that tended not to reported include those pertaining to study sample size (item 2 sub-item b [32%]), eligibility (item 3 sub-item a [33.3%] and b [41.1%]), blinding (item 5) (76.8%), results (sub-item 10 b 57.0%), animal care and monitoring (item 16 sub-item c [44.8%]), interpretation/scientific implications (item 17 sub-item b) (49.7%), generalisability/translation (item 18 [33.6%]), protocol registration (item 19 [35.2%]), and data access (item 20 [54.9%]). Conversely, adequate reporting included the following: study design (item 1 sub-item a [86.6%] and b [97.1%]), sample size (item 2 sub-item a [85.2%]), randomization (item 4 sub-item a [64.5%] and b [77.3%]), outcome measure (item 6 a [100.0%] and b [97.2%], statistical methods (item 7 a [79.7%] and b [89.3%]), experimental procedures (item a [98.2%], b [97.9%], c [59.3%] and d [99.3%]), results (item 10 sub-item a [99.7%]), abstract (item 11 [99.0%]), objectives (item 13 [99.5%]), ethical statement (item 17 [71.1%]) and declaration of interest (item 21 sub-item a [89.3%] and b [93.9%]) and housing and husbandry (item 15) (Fig. 2).

**Table 2.** Reporting adequacy of each ARRIVE 2.0 guideline item (*N* = 384).

ARRIVE 2.0 checklist item	Not reported *N* (%)	Inadequate reporting *N* (%)	Adequate reporting *N* (%)
**1. Study design**			
a. The groups being compared, including control groups. If no control group has been used, the rationale should be stated.	38 (9.9)	15 (3.9)	331 (86.2)
b. The experimental unit (e.g. a single animal, litter, or cage of animals).	1 (0.3)	10 (2.6)	373 (97.1)
**2. Sample size**			
a. Specify the exact number of experimental units allocated to each group, and the total number in each experiment. Also indicate the total number of animals used.	30 (7.8)	21 (7.0)	327 (85.2)
b. Explain how the sample size was decided. Provide details of any a priori sample size calculation, if done.	123 (32.0)	175 (45.6)	86 (22.4)
**3. Inclusion and exclusion criteria**			
a. Describe any criteria used for including and excluding animals (or experimental units) during the experiment, and data points during the analysis. Specify if these criteria were established a priori. If no criteria were set, state this explicitly.	128 (33.3)	150 (39.1)	106 (27.6)
b. For each experimental group, report any animals, experimental units or data points not included in the analysis and explain why. If there were no exclusions, state so	158 (41.1)	139 (36.2)	87 (22.7)
c. For each analysis, report the exact value of *n* in each experimental group.	38 (9.9)	19 (4.9)	327 (85.2)
**4. Randomisation**			
a. State whether randomization was used to allocate experimental units to control and treatment groups. If done, provide the method used to generate the randomisation sequence.	125 (32.6)	11 (2.9)	248 (64.5)
b. Describe the strategy used to minimize potential confounders such as the order of treatments and measurements, or animal/cage location. If confounders were not controlled, state this explicitly.	5 (1.3)	82 (21.4)	297 (77.3)
**5. Blinding**			
Describe who was aware of the group allocation at the different stages of the experiment (during the allocation, the conduct of the experiment, the outcome assessment, and the data analysis).	295 (76.8)	3 (0.8)	86 (22.4)
**6. Outcome measures**			
a. Clearly define all outcome measures assessed (e.g. cell death, molecular markers, or behavioural changes).	0 (0.0)	0 (0.0)	384 (100.0)
b. For hypothesis-testing studies, specify the primary outcome measure, i.e. the outcome measure that was used to determine the sample size.	2 (0.5)	9 (2.3)	373 (97.2)
**7. Statistical methods**			
a. Provide details of the statistical methods used for each analysis, including software used.	27 (7.0)	51 (13.3)	306 (79.7)
b. Describe any methods used to assess whether the data met the assumptions of the statistical approach, and what was done if the assumptions were not met.	3 (0.8)	38 (9.9)	343 (89.3)
**8. Experimental animals**			
a. Provide species-appropriate details of the animals used, including species, strain and substrain, sex, age or developmental stage, and, if relevant, weight.	3 (0.8)	9 (2.3)	372 (96.9)
b. Provide further relevant information on the provenance of animals, health/immune status, genetic modification status, genotype, and any previous procedures.	65 (16.9)	197 (51.3)	122 (31.8)
**9. Experimental procedures**			
For each experimental group, including controls, describe the procedures in enough detail to allow others to replicate them, including: a. What was done, how it was done and what was used.	1 (0.3)	6 (1.5)	377 (98.2)
b. When and how often.	2 (0.5)	6 (1.6)	376 (97.9)
c. Where (including detail of any acclimatization periods).	79 (20.6)	77 (20.1)	228 (59.3)
d. Why (provide rationale for procedures).	0 (0.0)	3 (0.7)	381 (99.3)
**10.Results**			
For each experiment conducted, including independent replications, report: a. Summary/descriptive statistics for each experimental group, with a measure of variability where applicable (e.g. mean and SD, or median and range).	0 (0.0)	1 (0.3)	383 (99.7)
b. If applicable, the effect size with a confidence interval.	219 (57.0)	64 (16.7)	101 (26.3)
**11. Abstract**			
Provide an accurate summary of the research objectives, animal species, strain and sex, key methods, principal findings, and study conclusions	2 (0.5)	2 (0.5)	380 (99.0)
**12. Background**			
a. Include sufficient scientific background to understand the rationale and context for the study, and explain the experimental approach.	0 (0.0)	5 (1.3)	379 (98.7)
b. Explain how the animal species and model used address the scientific objectives and, where appropriate, the relevance to human biology.	2 (0.5)	22 (5.7)	360 (93.8)
**13. Objectives**			
Clearly describe the research question, research objectives and, where appropriate, specific hypotheses being tested	0 (0.0)	2 (0.5)	382 (99.5)
**14. Ethical statement**			
Provide the name of the ethical review committee or equivalent that has approved the use of animals in this study, and any relevant licence or protocol numbers (if applicable). If ethical approval was not sought or granted, provide a justification.	102 (26.6)	9 (2.3)	273 (71.1)
**15. Housing and husbandry**			
Provide details of housing and husbandry conditions, including any environmental enrichment.	93 (24.2)	55 (14.3)	236 (61.5)
**16. Animal care and monitoring**			
a. Describe any interventions or steps taken in the experimental protocols to reduce pain, suffering and distress.	61 (15.9)	58 (15.1)	265 (69.0)
b. Report any expected or unexpected adverse events.	72 (18.8)	127 (33.1)	185 (48.1)
c. Describe the humane endpoints established for the study, the signs that were monitored and the frequency of monitoring. If the study did not have humane endpoints, state this.	172 (44.8)	113 (29.4)	99 (25.8)
**17. Interpretation/Scientific implications**			
a. Interpret the results, taking into account the study objectives and hypotheses, current theory and other relevant studies in the literature.	1 (0.3)	0 (0.0)	383 (99.7)
b. Comment on the study limitations including potential sources of bias, limitations of the animal model, and imprecision associated with the results.	191 (49.7)	42 (10.9)	151 (39.4)
**18. Generalisability/translation**			
Comment on whether, and how, the findings of this study are likely to generalize to other species or experimental conditions, including any relevance to human biology (where appropriate).	129 (33.6)	90 (23.4)	165 (43.0)
**19. Protocol registration**			
Provide a statement indicating whether a protocol (including the research question, key design features, and analysis plan) was prepared before the study, and if and where this protocol was registered.	135 (35.2)	61 (15.9)	188 (48.9)
**20. Data access**			
Provide a statement describing if and where study data are available.	211 (54.9)	44 (11.5)	129 (33.6)
**21. Declaration of interest**			
a. Declare any potential conflicts of interest, including financial and non-financial. If none exist, this should be stated.	40 (10.4)	61.(0.3)	343 (89.3)
b. List all funding sources (including grant identifier) and the role of the funder(s) in the design, analysis and reporting of the study.	50 (13.0)	12 (3.1)	322 (93.9)

The mean score for the sample was 57.9 (SD 6.7 and range 34–74). The mean score for the essential and recommended set items was 34.2 (SD 64.6 and range 17–44) and 23.8 (SD 3.5, range 13–32), respectively. Between journal titles, the mean score varied but interpretation of these scores needs to consider the number of studies published in each journal. For instance, the journal ACS Biomaterials Science & Engineering, only published one study which scored a mean score of 63. In contrast, American Journal of Orthodontics and Dentofacial Orthopaedics published 29 studies and the mean score was 60.9 (Supplementary Table III).

Table 3 shows the estimates, 95% confidence intervals and *P*-values for the effect of study characteristics on the ARRIVE 2.0 score. There were no associations between score and study characteristics except a weak association for year of publication with a small improvement over time: 0.41 for each additional year which translates to an average score increase by 2 units from the first to the last year of publication of the included studies.

**Table 3.** Estimates, 95% confidence intervals and p-values for the effect of study characteristics and ARRIVE 2.0 summary score.

Study characteristics		Coef.	*P*-value
Year of publication	2018	Reference	
	2019	0.46 (−1.93, 2.84)	.71
	2020	1.01 (−1.12, 3.14)	.35
	2021	1.11 (−1.15, 3.38)	.33
	2022	1.95 (−0.19, 4.09)	.07
	2023	1.70 (−1.06, 4.47)	.23
Continent of corresponding author	Europe	Reference	
	Americas	−0.76(−3.26, 1.74)	.55
	Asia and others	−1.34 (−3.48, 0.79)	.22
Centre	Single	Reference	
	Multi	0.15 (−1.53, 1.83)	.86
Number of authors	Per unit	−0.18 (−0.41, 0.05)	.13

## Discussion

The ARRIVE guidelines were introduced to improve the reporting of animal studies [15]. It is important to recognize that this distinct from assessing the quality of a study [8]. However, there are also wider and more ethical considerations as by improving reporting, the guidelines aim to improve the quality of life of animals, quality of animal experiments, and reduce the need for animals to be used in research [18]. Within this sample of animal studies published in the wider literature, the adequate reporting of certain ARRIVE 2.0 items was lacking in particular domains. These findings support the notion that reporting of key aspects in animal studies acts as one of the most common barriers against study reproducibility [19, 20]. Items that tended not to be reported include those pertaining to study sample size, eligibility, methods to reduce bias such as blinding, interpretation/scientific implications, and data access. Parallels can be drawn from previous studies which have reported similar findings [16, 17]. However, it should be borne in mind that these studies assessed reporting adherence in relation to the original ARRIVE guidelines. Although blinding is an important methodological step to reduce bias, its applicability to orthodontic *in-vitro* animal model studies needs to be considered for each study. For instance, PCR analysis of oral tissue may not require the need for blinding of the observer.

In almost a third of studies, there was a lack of clear reporting of the priori sample size calculation. It is well established that studies with animals tend to have small samples [21, 22]. When the sample is small, questions are raised as to the ability of the study to detect differences between the intervention or control/alternative intervention groups. The issue is further compounded by the lack of appropriate power/sample size calculations. In this scenario, the risk of detecting a Type I error (false positives) against a null hypothesis of no treatment effect is increased. Therefore, these studies will be prone to reporting overestimated treatment effects [12]. This has been highlighted in an interesting study, where the authors compared the reported treatment effects in animal and human studies for the non-surgical and surgical management of peri-implantitis and mucositis. Although not significant, compared to human trials larger treatment effects were reported in animal studies. The authors observed a large degree of heterogeneity between studies which hindered direct comparison and that animal studies were poorly reported [12].

Although not statistically significant, there appeared to be an improvement in reporting of animal studies in relation to the ARRIVE guidelines across the study timeframe. Additionally, the mean score for the essential set (34.2) of items was higher than the recommended set (23.8). This may be an indication of authors prioritizing the reporting on the essential set items. However, the variation in reporting items in both the essential and recommended set may reflect authors reporting items which they perceive relevant/important to their study.

The lack of adequate reporting of animal studies has been attributed to a lack awareness by researchers of the ARRIVE guidelines [18]. Furthermore, regional influences have been postulated as a barrier to widespread global adoption of the ARRIVE guidelines, which were devised in the United Kingdom [23]. Regardless, it is evident that recommendations to improve the reporting adherence of orthodontic studies using animal models in relation to the ARRIVE 2.0 guidelines are required. These should be directed at all stakeholders including journal editors, authors of studies, peer reviewers, and institutions [24]. Measures that can be undertaken by journal editors include insisting upon submission of a reporting checklist with the manuscript and removal of article word restrictions to allow researchers to fully describe all sections [23]. Journal editors should note that the active implementation of reporting checklists has been reported to result in improved reporting of studies such as Randomised Clinical Trials within orthodontics [25]. To further raise awareness, both journal editors and authors should be signposted to the Equator Network (www.equator-network.org) which aims to enhance the quality and transparency of health research.

The ARRIVE 2.0 guidelines replaced the initial guidelines published in 2010. It may be the case that our results represent some degree of underestimation of the reporting adequacy of the checklist items, as researchers may have been familiar with the initial version of the reporting guideline. In this investigation, animal studies relevant to the speciality of orthodontics published in the wider literature were analysed. This approach was adopted as it is more common for orthodontic studies that utilize animal models to be published in non- orthodontic journals. This is supported by the fact that an initial search of the top five orthodontic impact factor journals only identified 81 studies that met the study eligibility criteria. To reduce any subjectivity, the applicability of the study to the orthodontic speciality was agreed by discussion between two assessors. The possible introduction of bias into the current study maybe further increased by exclusion of non-English studies. However, it should be considered that our aim was to report a baseline of the general standard of reporting which shows there is a lack of adherence to certain items of the ARRIVE 2.0 guidelines.

Measures to reduce bias undertaken in this study were independent and duplicate screening and selection of studies, pre-piloting prior to data extraction, and referring directly to the description of each ARRIVE 2.0 guideline item to ensure consistency in the interpretation of each item during data extraction. Articles published approximately 3 years after the introduction of the latest version of the guidelines were included. We also included articles published 2 years before the introduction of the latest guideline to allow us to observe any trends in reporting. However, it could be argued that an adequate time period has not expired to allow the acceptance and awareness of the ARRIVE 2.0 guidelines by journal editors and researchers. This could be the hypothesis of future investigations.

## Conclusions

The reporting of animal studies relevant to the speciality of orthodontics are sub-optimal in relation to the ARRIVE 2.0 guidelines. There was a tendency for the non-reporting of items pertaining to study sample size, eligibility, methods to reduce bias, and interpretation/scientific implications. Greater awareness and reporting adherence to the ARRIVE 2.0 guidelines is required to reduce research waste involving animal models.

## Supplementary Material

cjae032_suppl_Supplementary_Material_S1

cjae032_suppl_Supplementary_Material_S2

cjae032_suppl_Supplementary_Material_S3

## Data Availability

The data underlying this article are available in the article and in its online supplementary material.
