# Supplementary material for: Still to ARRIVE at adequate reporting of orthodontic studies involving animal models
Source: Eur J Orthod. 2024 Jul 15;46(4):cjae032. doi: 10.1093/ejo/cjae032 (PMC11247523; doi:10.1093/ejo/cjae032)
Supplement: cjae032_suppl_Supplementary_Material_S2 [file cjae032_suppl_supplementary_material_s2.docx]

| **Journal Title** | **N (%)** |
| --- | --- |
| ACS Biomaterials Science & Engineering | 1 (0.3%) |
| ACS Nano | 2 (0.5%) |
| Acta Biomateriala | 1 (0.3%) |
| ACTA CIRÚRGICA BRASILEIRA | 1 (0.3%) |
| Ahead of Print | 1 (0.3%) |
| Alcohol | 1 (0.3%) |
| American Journal of Orthodontics and Dentofacial Orthopedics | 29 (7.6%) |
| Angle Orthodontist | 13 (3.4%) |
| Annals of Anatomy | 5 (1.3%) |
| Archives of Biochemistry and Biophysics | 1 (0.3%) |
| Archives of Oral Biology | 25 (6.5%) |
| Association of Avian Veterinatians Australasian Committee Ltd Annual Conference | 1 (0.3%) |
| Australian Endodontic Journal | 1 (0.3%) |
| Bioelectromagnetics | 1 (0.3%) |
| BioMed Research International | 5 (1.3%) |
| Biomedical Engineering Society | 1 (0.3%) |
| Biomedicine& Pharmacotherapy | 2 (0.5%) |
| Biomolecules & Biomedicine | 1 (0.3%) |
| Bioscience Reports | 1 (0.3%) |
| BMC Complementary Medicine and Therapies | 1 (0.3%) |
| BMC Oral Health | 5 (1.3%) |
| Bone | 7 (1.8%) |
| Brain and Behavior | 1 (0.3%) |
| Brain Research | 1 (0.3%) |
| Brazil Oral Research | 1 (0.3%) |
| Brazilian Dental Journal | 1 (0.3%) |
| CELL CYCLE | 1 (0.3%) |
| Cell Proliferation | 1 (0.3%) |
| Cells | 2 (0.5%) |
| Cells 2022 | 1 (0.3%) |
| Cellular Signaling | 2 (0.5%) |
| Chinese Journal of Dental Research | 1 (0.3%) |
| Cirugía y Cirujanos | 1 (0.3%) |
| Clinical and Experimental Dental Research | 3 (0.8%) |
| Clinical Oral Investigations | 12 (3.1%) |
| Computational and Mathematical Methods in Medicine | 1 (0.3%) |
| Cytokine | 1 (0.3%) |
| Dental Materials Journal | 4 (1.0%) |
| Dental Press Jounral of Orthodontics | 1 (0.3%) |
| Dental Press Journal of Orthodontics | 2 (0.5%) |
| Dental Traumatology | 1 (0.3%) |
| Drug Design, Development and Therapy | 3 (0.8%) |
| Equine Veterinary Journal | 1 (0.3%) |
| European Journal of Oral Sciences | 3 (0.8%) |
| European Journal of Orthodontics | 18 (4.7%) |
| European Review for Medical and Pharmacological Sciences | 2 (0.5%) |
| Experimental & Molecular Medicine | 1 (0.3%) |
| Experimental Cell Research | 1 (0.3%) |
| F1000 Research | 2 (0.5%) |
| Folia Medica | 2 (0.5%) |
| Folia Morphologica | 1 (0.3%) |
| Free Radical Biology and Medicine | 1 (0.3%) |
| Frontiers in Immunology | 2 (0.5%) |
| Functional & Integrative Genomics | 1 (0.3%) |
| Head & Face Medicine | 4 (1.0%) |
| Hindawi | 1 (0.3%) |
| Histology and Histopathology | 1 (0.3%) |
| in vivo | 1 (0.3%) |
| In vivo | 1 (0.3%) |
| Inflammation | 2 (0.5%) |
| International Endodontic Journal | 1 (0.3%) |
| International Immunopharmacology | 2 (0.5%) |
| International Journal for Numerical Methods in Engineering | 1 (0.3%) |
| International Journal of Molecular Sciences | 7 (1.8%) |
| International Journal of Oral Maxillofacial surgery | 1 (0.3%) |
| International journal of oral Science | 1 (0.3%) |
| International Journal of Oral Science | 8 (2.1%) |
| International Orthodontics | 4 (1.0%) |
| Japanese Association for Laboratory Animal Science | 1 (0.3%) |
| Journal of Applied Oral Science | 3 (0.8%) |
| Journal of Biomechanics | 2 (0.5%) |
| Journal of Bone and Mineral Metabolism | 2 (0.5%) |
| Journal of Cellular Biochemistry | 2 (0.5%) |
| Journal of Cellular Molecular Medicine | 1 (0.3%) |
| Journal of Cellular Physiology | 3 (0.8%) |
| Journal of Clinical Periodontology | 2 (0.5%) |
| Journal of Dental Research | 9 (2.3%) |
| Journal of Diabetes Investigation | 1 (0.3%) |
| Journal of Fluorescence | 1 (0.3%) |
| Journal of Healthcare Engineering | 1 (0.3%) |
| Journal of Immunology Research | 1 (0.3%) |
| Journal of International Medical Research | 1 (0.3%) |
| Journal of Med Primatol | 1 (0.3%) |
| Journal of Medicine and Life | 1 (0.3%) |
| Journal of Molcu | 1 (0.3%) |
| Journal of Molecular Histology | 2 (0.5%) |
| Journal of Musculoskeletal Neuronal internations | 2 (0.5%) |
| Journal of Oleo Science | 1 (0.3%) |
| Journal of Oral Biosciences | 1 (0.3%) |
| Journal of Oral Rehabilitation | 1 (0.3%) |
| Journal of Oral Science | 1 (0.3%) |
| Journal of Oroal Maxillofacial Surgery | 1 (0.3%) |
| Journal of Orofacial Orthopedics | 7 (1.8%) |
| Journal of Orthopaedic Surgery | 1 (0.3%) |
| Journal of Peridontal Research | 5 (1.3%) |
| Journal of Peridontology | 1 (0.3%) |
| Journal of Periodontal Research | 2 (0.5%) |
| Journal of Periodontology | 3 (0.8%) |
| Journal of Photochemistry & Photobiology | 1 (0.3%) |
| Journal of Proteomics | 1 (0.3%) |
| Journal of Stomatology Oral & Maxillofacial Surgery | 1 (0.3%) |
| Journal of the Formosan Medical Association | 1 (0.3%) |
| Journal of the Mechanical behaviour of biomedical materials | 2 (0.5%) |
| Journal of the World Federation of Orthodontists | 1 (0.3%) |
| Journal of Thermal Biology | 1 (0.3%) |
| Journal of Veterinary Dentistry | 3 (0.8%) |
| Lasers in Medical Science | 4 (1.0%) |
| Life Sciences | 1 (0.3%) |
| Medical Science Monitor | 1 (0.3%) |
| Medicina | 1 (0.3%) |
| Microscopy Research & Technique | 1 (0.3%) |
| Minerva Medica | 1 (0.3%) |
| Molecular Medicine Reports | 5 (1.3%) |
| Molecular Pain | 1 (0.3%) |
| Molecules | 1 (0.3%) |
| Neuroscience Research | 2 (0.5%) |
| Nutrients | 2 (0.5%) |
| Odontology | 2 (0.5%) |
| Oral and Maxillofacial Surgery | 1 (0.3%) |
| Oral Diseases | 6 (1.6%) |
| Original Research Orthodontics | 2 (0.5%) |
| Orthodontics & Craniofacial research | 14 (3.6%) |
| Pain Research and Management | 1 (0.3%) |
| Photobiomodulation, Photomedicine, and Laser Surgery | 1 (0.3%) |
| Photochemistry and Photobiology | 3 (0.8%) |
| Photomedicine and Laser Surgery | 1 (0.3%) |
| PLOS One | 1 (0.3%) |
| PLOS ONE | 4 (1.0%) |
| Progress in orthodontics | 6 (1.6%) |
| Scientific Reports - nature research | 18 (4.7%) |
| Stem Cell Reseach & Therapy | 4 (1.0%) |
| Stem Cells and Development | 1 (0.3%) |
| Stress | 1 (0.3%) |
| The Angle Orthodontist | 1 (0.3%) |
| The FASEB Journal | 4 (1.0%) |
| The Journal of Adhesive Dentistry | 1 (0.3%) |
| The Journal of Contemporary Dental Practice | 1 (0.3%) |
| Therapeutic Delivery | 1 (0.3%) |
| Tissue Engineering & Regenerative Medicine International Society | 1 (0.3%) |

Supplementary Table II Journal titles (N=384)
