# Supplementary material for: Still to ARRIVE at adequate reporting of orthodontic studies involving animal models
Source: Eur J Orthod. 2024 Jul 15;46(4):cjae032. doi: 10.1093/ejo/cjae032 (PMC11247523; doi:10.1093/ejo/cjae032)
Supplement: cjae032_suppl_Supplementary_Material_S3 [file cjae032_suppl_supplementary_material_s3.docx]

| **Journal title** | **N** | **Mean** |
| --- | --- | --- |
|  |  |  |
| ACS Biomaterials | 1 | 63 |
| ACS Nano | 2 | 56.5 |
| ACTA CIRÚRGICA B | 1 | 63 |
| Acta Biomaterial | 1 | 57 |
| Ahead of Print | 1 | 60 |
| Alcohol | 1 | 65 |
| American Journal | 29 | 60.93103 |
| Angle Orthodonti | 13 | 52.30769 |
| Annals of Anatom | 5 | 61.8 |
| Archives of Bioc | 1 | 46 |
| Archives of Oral | 25 | 59.2 |
| Association of A | 1 | 41 |
| Australian Endod | 1 | 59 |
| BMC Complementar | 1 | 53 |
| BMC Oral Health | 5 | 62.6 |
| BioMed Research | 5 | 55.2 |
| Bioelectromagnet | 1 | 65 |
| Biomedical Engin | 1 | 61 |
| Biomedicine& Pha | 2 | 51.5 |
| Biomolecules & B | 1 | 64 |
| Bioscience Repor | 1 | 54 |
| Bone | 7 | 56.14286 |
| Brain Research | 1 | 42 |
| Brain and Behavi | 1 | 62 |
| Brazil Oral Rese | 1 | 57 |
| Brazilian Dental | 1 | 61 |
| CELL CYCLE | 1 | 60 |
| Cell Proliferati | 1 | 54 |
| Cells | 1 | 58 |
| Cells | 1 | 55 |
| Cells 2022 | 1 | 57 |
| Cellular Signali | 2 | 57 |
| Chinese Journal | 1 | 58 |
| Cirugía y Ciruja | 1 | 49 |
| Clinical Oral In | 12 | 60.75 |
| Clinical and Exp | 3 | 62 |
| Computational an | 1 | 48 |
| Cytokine | 1 | 66 |
| Dental Materials | 4 | 50.5 |
| Dental Press Jou | 1 | 38 |
| Dental Press Jou | 2 | 58.5 |
| Dental Traumatol | 1 | 66 |
| Drug Design, Dev | 3 | 62 |
| Equine Veterinar | 1 | 54 |
| European Journal | 3 | 60 |
| European Journal | 18 | 61.33333 |
| European Review | 2 | 62.5 |
| Experimental & M | 1 | 53 |
| Experimental Cel | 1 | 59 |
| F1000 Research | 2 | 71.5 |
| Folia Medica | 2 | 60 |
| Folia Morphologi | 1 | 60 |
| Free Radical Bio | 1 | 61 |
| Frontiers in Imm | 2 | 63.5 |
| Functional & Int | 1 | 53 |
| Head & Face Medi | 4 | 61.75 |
| Hindawi | 1 | 68 |
| Histology and Hi | 1 | 56 |
| In vivo | 1 | 62 |
| Inflammation | 2 | 55.5 |
| International En | 1 | 51 |
| International Im | 2 | 57.5 |
| International Jo | 1 | 49 |
| International Jo | 7 | 55.57143 |
| International Jo | 1 | 65 |
| International Jo | 2 | 63 |
| International Jo | 6 | 58 |
| International Or | 4 | 59 |
| International jo | 1 | 58 |
| Japanese Associa | 1 | 59 |
| Journal of Appli | 3 | 57.33333 |
| Journal of Biome | 2 | 61 |
| Journal of Bone | 2 | 61.5 |
| Journal of Cellu | 2 | 55 |
| Journal of Cellu | 1 | 59 |
| Journal of Cellu | 3 | 53.66667 |
| Journal of Clini | 2 | 61 |
| Journal of Denta | 9 | 51.11111 |
| Journal of Diabe | 1 | 43 |
| Journal of Fluor | 1 | 46 |
| Journal of Healt | 1 | 59 |
| Journal of Immun | 1 | 45 |
| Journal of Inter | 1 | 60 |
| Journal of Med P | 1 | 38 |
| Journal of Medic | 1 | 58 |
| Journal of Molcu | 1 | 52 |
| Journal of Molec | 2 | 52.5 |
| Journal of Muscu | 2 | 51.5 |
| Journal of Oleo | 1 | 36 |
| Journal of Oral | 1 | 54 |
| Journal of Oral | 1 | 63 |
| Journal of Oral | 1 | 54 |
| Journal of Oroal | 1 | 58 |
| Journal of Orofa | 7 | 59.85714 |
| Journal of Ortho | 1 | 63 |
| Journal of Perid | 5 | 55 |
| Journal of Perid | 1 | 63 |
| Journal of Perio | 2 | 50.5 |
| Journal of Perio | 3 | 57.33333 |
| Journal of Photo | 1 | 60 |
| Journal of Prote | 1 | 73 |
| Journal of Stoma | 1 | 69 |
| Journal of Therm | 1 | 53 |
| Journal of Veter | 3 | 46.66667 |
| Journal of the F | 1 | 54 |
| Journal of the M | 2 | 47.5 |
| Journal of the W | 1 | 61 |
| Lasers in Medica | 4 | 59 |
| Life Sciences | 1 | 56 |
| Medical Science | 1 | 58 |
| Medicina | 1 | 66 |
| Microscopy Resea | 1 | 59 |
| Minerva Medica | 1 | 44 |
| Molecular Medici | 5 | 59 |
| Molecular Pain | 1 | 57 |
| Molecules | 1 | 67 |
| Neuroscience Res | 2 | 58.5 |
| Nutrients | 2 | 64.5 |
| Odontology | 2 | 60.5 |
| Oral Diseases | 6 | 56.66667 |
| Oral and Maxillo | 1 | 54 |
| Original Researc | 2 | 55 |
| Orthodontics & C | 14 | 57.64286 |
| PLOS ONE | 4 | 52 |
| PLOS One | 1 | 60 |
| Pain Research an | 1 | 64 |
| Photobiomodulati | 1 | 56 |
| Photochemistry a | 3 | 61.33333 |
| Photomedicine an | 1 | 57 |
| Progress in orth | 6 | 59 |
| Scientific Repor | 18 | 61.22222 |
| Stem Cell Reseac | 4 | 53 |
| Stem Cells and D | 1 | 61 |
| Stress | 1 | 62 |
| The Angle Orthod | 1 | 58 |
| The FASEB Journa | 4 | 57 |
| The Journal of A | 1 | 60 |
| The Journal of C | 1 | 51 |
| Therapeutic Deli | 1 | 66 |
| Tissue Engineeri | 1 | 57 |
| in vivo | 1 | 57 |
|  |  |  |
| Total | 384 | 57.92448 |
|  |  |  |

Supplementary Table III
